# Epileptic brain imaging by source localization CLARA supported by ictal-based semiology and VEEG in resource-limited settings

**DOI:** 10.3389/fninf.2025.1661617

**Published:** 2025-08-29

**Authors:** Amir F. Al-Bakri, Ahmed Tahseen Muslim, Moneer K. Faraj, Wamedh Esam Matti, Radana Vilimkova Kahankova, Dariusz Mikolajewski, Waldemar Karwowski, Aleksandra Kawala-Sterniuk

**Affiliations:** ^1^Department of Biomedical Engineering, College of Engineering, University of Babylon, Babylon, Iraq; ^2^Al-Witri Hospital for Neurosciences, Baghdad, Iraq; ^3^Department of Clinical Neurophysiology, College of Medicine, University of Baghdad, Baghdad, Iraq; ^4^Faculty of Electrical Engineering, Automatic Control and Informatics, Opole University of Technology, Opole, Poland; ^5^Department of Cybernetics and Biomedical Engineering, Faculty of Electrical Engineering and Computer Science, VSB–Technical University of Ostrava, Ostrava, Czechia; ^6^Institute of Computer Science, Kazimierz Wielki University in Bydgoszcz, Bydgoszcz, Poland; ^7^Neuropsychological Research Unit, 2nd Clinic of the Psychiatry and Psychiatric Rehabilitation, Medical University in Lublin, Lublin, Poland; ^8^Computational Neuroergonomics Laboratory, Department of Industrial Engineering and Management Systems, University of Central Florida, Orlando, FL, United States; ^9^Department of Artificial Intelligence, Faculty of Information and Communication Technology, Wroclaw University of Science and Technology, Wroclaw, Poland

**Keywords:** epilepsy, brain signals, VEEG, brain imaging, classical LORETA analysis recursively applied (CLARA)

## Abstract

**Introduction:**

Accurate localization of the epileptogenic zone is essential for surgical treatment of drug-resistant epilepsy. Standard presurgical evaluations rely on multimodal neuroimaging techniques, but these may be limited by availability and interpretive challenges. This study aimed to assess the concordance between zones identified by ictal semiology and a novel distributed electrical source localization technique, CLARA, and to evaluate their impact on postsurgical outcomes.

**Methods:**

This retrospective study included 16 patients with at least three recorded seizures. Ictal semiology was analyzed subjectively using video electroencephalography (VEEG) by a multidisciplinary team of neurologists, neurophysiologists, and radiologists, who determined the presumed epileptogenic zone at the lobar level. CLARA was subsequently applied to identify the computed zone based on ictal and/or interictal biomarker activities. The concordance between the presumed and computed zones was assessed qualitatively. Postsurgical outcomes were examined in relation to the extent of resection of the CLARA-defined zones.

**Results:**

Among thirteen patients with sufficient data for analysis, qualitative comparison showed 77% concordance and 23% partial concordance between the presumed and computed zones. Postsurgical follow-up revealed seizure freedom in one patient with cavernoma following complete resection of the CLARA-defined zone. In contrast, patients with incomplete resection of this region continued to experience seizures.

**Discussion:**

The findings support the potential value of CLARA as an adjunctive neuroimaging technique in the presurgical evaluation of epilepsy. By providing an additional layer of verification, CLARA may improve the accuracy of epileptogenic zone localization when used alongside established modalities such as PET, SPECT, fMRI, and MRI. Its adaptability and lower resource requirements suggest particular utility in centers with limited access to advanced medical equipment and specialized personnel. Broader implementation of CLARA could enhance presurgical decision-making and contribute to improved surgical outcomes for epilepsy patients.

## 1 Introduction

Epilepsy affects ~1% of the global population, with around 30% of patients being resistant to medical treatment (Sander, 2003). For those with drug-resistant focal epilepsy, surgical removal of the seizure-generating area is often an effective option (Kwan and Brodie, 2000). Successful identification and excision of this region can lead to favorable outcomes, with 30%–85% of patients achieving seizure freedom (Rosenow and Lüders, 2001; Kim et al., 2024).

Epilepsy is frequently associated with clinical signs supported by abnormal electrical activity originating in specific brain regions. These clinical signs, or semiology, can manifest as significant changes in motor or behavioral activity, with or without loss of consciousness (Eadie and Bladin, 2001). Semiology is valuable for determining the location of the symptomatogenic zone, which is typically close to the epileptogenic zone, and can achieve up to 74% lateralization and 77% localization accuracy (Chowdhury et al., 2021; Elwan et al., 2018). Relevant semiology is usually obtained through careful analysis of the patient's history, video telemetry, and electroencephalogram (EEG) recordings during multiple events (Frazzini et al., 2022; Hirfanoglu et al., 2007; Otárula and Schuele, 2024). However, interpreting semiology can be complex and requires expert clinicians for accurate diagnosis and presurgical evaluation (Foldvary-Schaefer and Unnwongse, 2011; Otárula and Schuele, 2024).

In addition to clinical presentation, a multimodal presurgical examination is crucial for determining the location and extent of the seizure origin. Standard approaches include structural and functional MRI, PET, SPECT, conventional EEG, video-EEG, and neuropsychological testing (Ryvlin et al., 2014; Barnova et al., 2023; Sharma et al., 2019; Rikir et al., 2020). Neuroimaging tools can localize the seizure onset zone with up to 80% accuracy, depending on whether epileptic lesions are present (Knowlton et al., 2008; Barnova et al., 2023). This is important because the sensitivity of scalp recordings is low, particularly in non-lesional or extra-temporal lobe epilepsy. Moreover, such recordings are typically analyzed visually, leading to subjective interpretations (Singh et al., 2022; Toole et al., 2019; Kawala-Sterniuk et al., 2021; Barnova et al., 2023). Despite these challenges, scalp studies remain valuable for source localization (Eom, 2023; Karikari and Koshechkin, 2023).

The findings from the aforementioned tools are somewhat subjective, relying heavily on the visual analysis and expertise of the diagnostician. Therefore, incorporating computational techniques to reduce subjectivity is necessary to enhance presurgical evaluation. Electrical source localization (ESL) is a non-invasive method that utilizes conventional brainwave recordings and applies empirical models of dipole and distributed source localization for imaging the epileptic brain (Plummer et al., 2008; Asadzadeh et al., 2020; Rikir et al., 2020). ESL employs interictal and ictal activity, such as spikes and slow delta waves, in conjunction with structural MRI, to determine the volumetric location of the seizure generator within a realistic head model for patients (Singh et al., 2022; Herrendorf et al., 2000; Michel and Brunet, 2019).

With the increase in the number of EEG channels, the sensitivity of ESL in localizing the epileptogenic focus can reach up to 94%, surpassing other imaging tools such as PET (up to 66%), SPECT (up to 64%), and MRI for lesional epilepsy (up to 76%) (Brodbeck et al., 2011; Duez et al., 2019; Brinkmann, 2024). Accurately determining and safely resecting the epileptogenic zone significantly enhances the likelihood of achieving seizure freedom for patients. In this study, we applied a novel neuroimaging ESL technique called Classic LORETA Recursively Applied (CLARA) (Irandoost et al., 2023) using BESA Research 7.1 software (Hoechstetter et al., 2010) and assessed its performance. This technique is intended as an additional neuroimaging tool to verify and enhance the presurgical evaluation conducted with standard tools at our hospital, such as semiology, MRI, and video-EEG, particularly for patients being considered for lobectomy (Kim et al., 2024; Rampp et al., 2024).

BESA is a reliable and user-friendly commercial software that offers several advanced ESL models, including Dipole, CLARA, LORETA, and Cortical-CLARA, each with distinct advantages (Iordanov et al., 2014, 2018):

- **Dipole model**—ideal for representing small clusters of active neurons, such as those in epileptic regions.
- **CLARA and LORETA**—suitable for modeling widespread brain activity, including both superficial and deep structures, making them effective for analyzing fundamental brain rhythms such as alpha waves.
- **Cortical-CLARA model**—focuses on estimating neural activity localized to the cortical surface.

For our analysis, which involves both epileptiform discharge activity and fundamental rhythms (e.g., alpha, beta), we chose CLARA over LORETA. CLARA outperforms LORETA by producing clearer source images with reduced blurring and better differentiation of closely located brain regions. It enhances spatial resolution by iteratively excluding low-activity regions, recalculating the source distribution, and retaining the areas with the strongest activation.

## 2 Materials and methods

In this study, 24-h video EEG recordings were monitored and analyzed by neurophysiologists and neurologists, whose expertise ensured a thorough examination of the data and provided critical insights into the neurophysiological aspects under investigation.

### 2.1 Data acquisition

Clinical data were obtained from 24-h video-EEG (VEEG) recordings of 16 patients diagnosed with epilepsy. These recordings were conducted retrospectively between 2021 and 2024 at the Epilepsy Monitoring Unit of Dr. Saad Al-Witry Hospital for Neurosciences in Baghdad, Iraq. Each recording was performed at a sampling rate of 256 Hz using 19 surface electrodes placed according to the international 10–20 system.

The clinical evaluation process involved both neurologists and neurophysiologists, who used VEEG recordings in combination with MRI scans to localize the symptomatogenic zone at the lobar level and define the seizure onset zone (SOZ). Subsequently, a biomedical engineering expert specializing in brain signal processing applied the CLARA method (Classical LORETA Analysis Recursively Applied) to further refine the localization of the epileptogenic zone.

All patient data were collected in full compliance with ethical and regulatory standards. Written informed consent was obtained from all participants prior to inclusion in the study. The research was conducted in accordance with the Declaration of Helsinki and received ethical approval from the Ministry of Health and Environment of Iraq, the Al-Russafa Health Directorate, and Dr. Saad Al-Witry Hospital for Neurosciences (Approval No. 2, dated July 14th, 2024; Protocol/Form Nos. 20240714 and 02/2021).

This study was conducted within a hospital setting specializing in adult neurology; accordingly, pediatric patients were not included. Additionally, patient selection was non-random and based on convenience-based criteria, for example, the inclusion of adult patients from our institution who were medicine-resistant and considered good candidates for lobectomy.

To remove artifacts, a 1 − 70 Hz band-pass filter was applied. All analyses were conducted retrospectively using BESA Research 7.1 (trial version) and the Natus EEG machine, with the option to export data in European Data Format (EDF). Although the initial plan was to test 16 patients with at least three clear seizures, only 14 were ultimately selected for analysis.

### 2.2 Criteria for patient and seizure feature detection

Epileptic features appearing during the ictal phases of seizure onset and/or epileptiform discharge activities occurring during the interictal phases were visually inspected and labeled after applying a common average montage. Only 14 patients, aged 16–45 years, who exhibited prominent epileptic characteristics in VEEG, clear semiology, and a clear MRI report were selected for CLARA analysis. These features varied among patients.

The following epilepsy characteristics were considered (Beniczky et al., 2013; Cabrerizo et al., 2011; Foldvary et al., 2001; Mattioli et al., 2022):

1. **Rhythmic activity**—delta, theta, and alpha waves.
2. **Paroxysmal fast activity**—rhythmic activity above 13 Hz, such as beta and gamma waves.
3. **Epileptiform discharge activity**—spikes and spike-wave complexes.

From the entire recording of each patient, at least three suitable 5-min EEG windows containing clear ictal and/or interictal features were extracted and used for the CLARA analyses.

### 2.3 Averaging the repetitive waveforms

Using BESA Research 7.1, an additional band-pass filter (0.5 − 30 Hz) was applied to the 5-min EEG windows. To compute the grand average of similar repetitive waveforms, the following steps, based on Ortiz-Mantilla et al. (2012), were followed:

1. **Main channel identification**—the main channel was selected through visual inspection, identifying the channel with the most prominent epileptic features (e.g., spikes, beta activity) and highest amplitude relative to others.
2. **Positive peaks marking and 2D voltage head distribution generation**—positive peaks in the selected channel were manually marked, and a 2D voltage head distribution was generated to visualize spatial voltage variations across all channels.
3. **Peaks with similar distribution labeling**—peaks with similar temporal and spatial distributions were grouped as **pattern 1**. Due to variability, strict pattern definitions were not always feasible; therefore, classification focused on visually similar peaks.
4. **Fixed time window definition**—a fixed time window was set around each peak to capture the waveform duration: ±12.5 ms for delta waves at 2 Hz, ±2.5 ms for alpha waves at 10 Hz, and custom windows for spikes based on their width.
5. **Repetitive waveform averaging across all channels**—the selected peaks were averaged across all channels to produce a grand average waveform, which was then used for source localization analysis.

### 2.4 Source localization analyses

Due to the unavailability of individual MRI images in the hospital database, an age-matched average MRI template provided by the BESA software was used to reconstruct a realistic head model.

**Figure 1:**
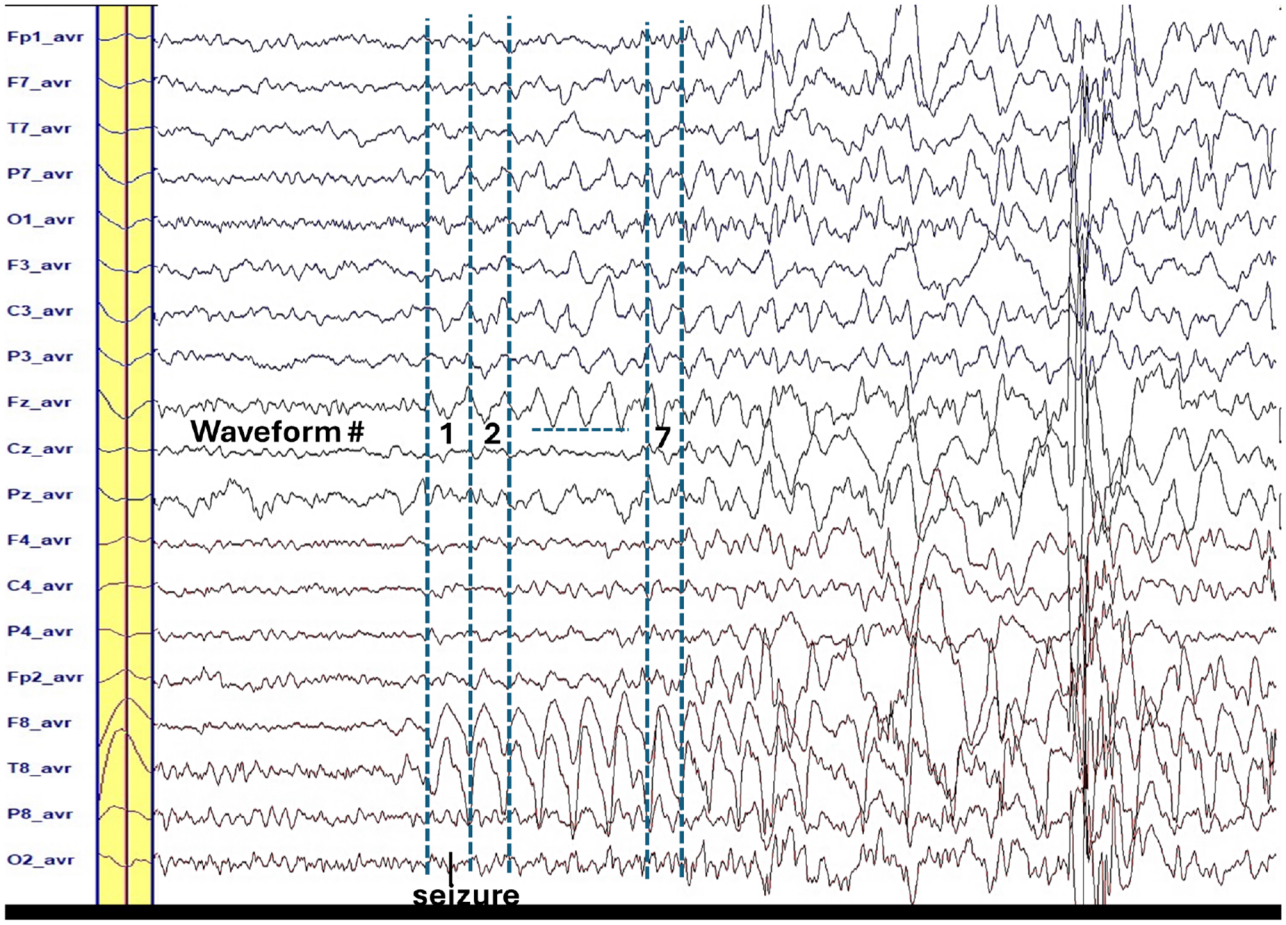
The yellow bar is the average waveform for the repetitive seven waveforms of the ictal slow wave (2 Hz). The peaks of these waveforms have similar 2-D voltage distribution (see Figure 2). Time window is ±60 ms around the peak that is the highest at the **T8-avr** channel.

**Figure 2:**
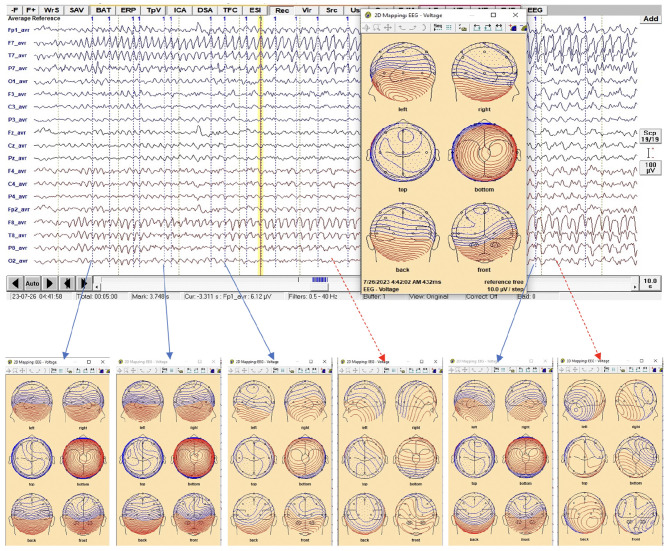
EEG trace during the ictal phase that has the epileptic feature which is alpha for this patient. The only peaks along the feature that provided similar 2-D voltage distribution were selected and labeled as **pattern 1**, where the 2-D mapping window is displayed when a right click is made at any time point on the EEG (yellow vertical line).

The average waveform, within its defined time window across channels, was selected for epileptic source localization analysis using BESA Research 7.1 (Figure 1). In Figure 2, examples of 2D voltage distributions for six peaks are shown. Peaks labeled as **pattern 1** (blue solid arrow) exhibited similar 2D mappings, while peaks with differing 2D mappings (red dashed arrow) were excluded from further analysis. Figures 1, 2 show representative ictal epileptic characteristics used in source localization for different patients. Only peaks with matching 2D voltage distributions were labeled as **pattern 1** for each characteristic.

**Figure 3:**
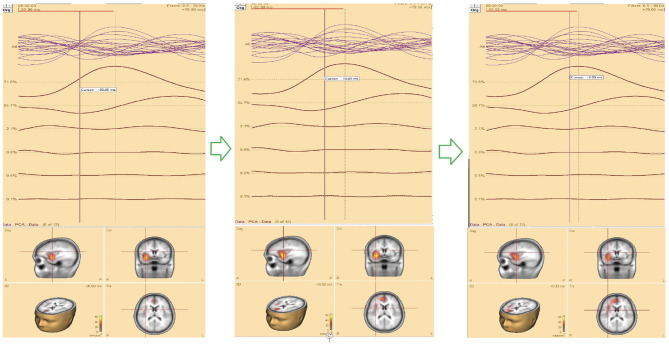
**(Top)** The upper trace is the butterfly of the average waveform of all channels (100 ms duration). The lower traces are the PCA components of the average waveform that are showed from high to low based on their contributed variances, where the first component has the highest variance (98.1%) when the rising part of the average waveform is selected (35 ms duration), and **(bottom)** Initial **SOZ** and its extension corresponding to different time. Left: only initial **SOZ** at temporal lobe (@*t* = −30 ms), middle: **SOZ** at temporal lobe and slightly extension into frontal lobe (@*t* = −18 ms), right: complete **SOZ** at temporal and frontal lobes (@*t* = −6 ms).

For source localization, the average waveform was co-registered with the age-appropriate MRI template. Principal component analysis (PCA) was then applied to the grand average waveform across channels to reduce dimensionality and identify the most significant components with the highest variance (Figure 3, top panel).

Theoretically, the rising segment of epileptic signals—from onset to maximum peak-contains the most critical information about onset activity. Analyzing this portion improves the accuracy of source localization (Ding et al., 2007; Lantz et al., 2003; Mălîia et al., 2016).

In this study, we refined and selected the rising segment of the average waveform, ensuring that the first PCA component accounted for ≥95% of the total variance. This approach minimized the residual signal between the modeled and observed EEG, thereby increasing the accuracy of focal area identification. The selected segment was then analyzed using the Classical LORETA Recursively Applied (CLARA) method (Iordanov et al., 2014, 2018) (see Figure 2).

One advantage of BESA Research 7.1 is its ability to navigate through the selected EEG signal (e.g., the rising segment) and identify the corresponding seizure onset zone (SOZ). This functionality was particularly valuable for pinpointing the initial SOZ and mapping its extension.

### 2.5 Epileptic zones concordance

Following presurgical epilepsy evaluation guidelines, the clinical findings from semiology, supported independently by VEEG and MRI, were obtained by specialists from different disciplines. The information from each specialty was then integrated to identify the candidate epileptic zone.

To qualitatively estimate the overlap between visually identified brain regions, we compared the presumed epileptic zone, determined by neurologists, neurophysiologists, and radiologists, with the computed epileptic zone obtained using CLARA (Tortora et al., 2022). The analysis focused on the four main cerebral lobes (frontal, temporal, parietal, occipital) without further subdivision.

The class of concordance was defined as follows:

1. **Concordant**: the presumed and computed epileptic zones overlapped entirely.
2. **Partially concordant**: the zones overlapped within the same region but were not fully aligned.
3. **Discordant-ipsilateral**: the zones were in different regions within the same hemisphere.
4. **Discordant-contralateral**: the zones were located in opposite hemispheres.
5. **Uninformative**: no localization could be determined.

Partially concordant refers to overlapping zones within the same region (e.g., temporal lobe), but not fully aligned. Discordant describes zones in different regions (e.g., frontal vs. temporal lobe). Ipsilateral means the zones are in the same hemisphere (left or right). Contralateral refers to zones in opposite hemispheres (left vs. right).

## 3 Results

The demographics, semiology, presumed and computed seizure onset zones (SOZ), and concordance classifications are summarized in Table 1. The table also lists seizure states (interictal and ictal) and identifies the EEG channels with the most significant epileptic features.

**Table 1:** Clinical data, presumed zone and computed zone supported by CLARA brain mapping, and concordance level.

**No**.	**Gender/ age**	**Ictal semiology verified by VEEG 24 h**	**Presumed epileptic zone (ictal symptomatogenic zone)—marked by neurologists and neurophysiologists**	**State/activity (positive channels)**	**Computed epileptic zone—determined with CLARA**	**Concordance**	**Brain mapping**
**P1**	**Male/45**	Focal: tonic seizures (brief fencing posture with extended right arm and flexed left arm)	Focal: frontal lobe	Ictal and interictal/theta and spikes (FP1, FP2, T7)	Focal: frontal and left temporal lobe	Partial concordant	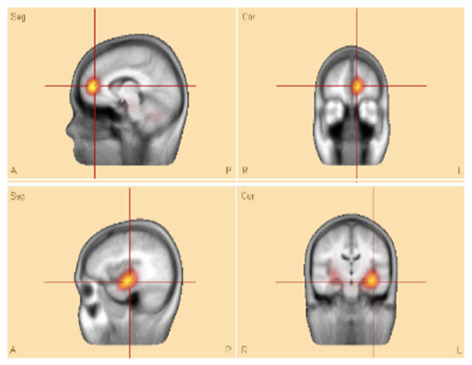
**P3**	**Male/36**	Hyper motor seizure followed by staring attack with lip smacking and mild automatism	Focal: frontal vs. temporal lobe	Ictal and interictal/theta and spikes (F7, F8, T7)	Focal: starting at frontal and extended into left temporal lobe	Concordant	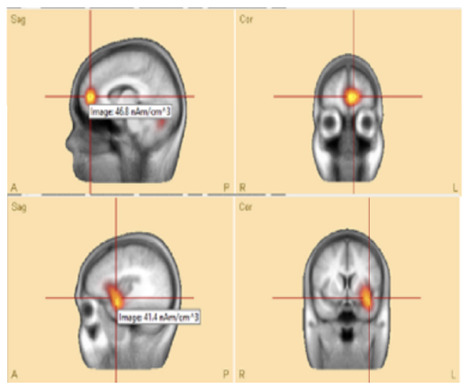
**P4**	**Male/21**	Tonic head and eyes deviation to right side with evolution to bilateral tonic clonic seizures	Focal: frontal vs. temporal lobe	Ictal and preictal/slow wave delta and spikes (FP1, FP2, F7, F8, T8, P8)	Focal: starting at bifrontal and extended into right temporal lobe	Concordant	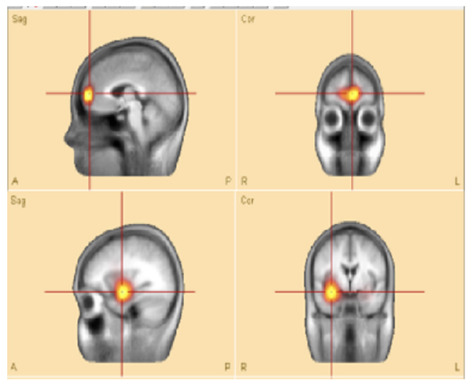
**P5**	**Male/33**	Hyper motor seizures followed by versive head and eyes deviation to left side	Multifocal: unknown lobe	Ictal/slow wave delta (almost all channels)	Multifocal: biparietal and left temporal lobes	Uninformaive	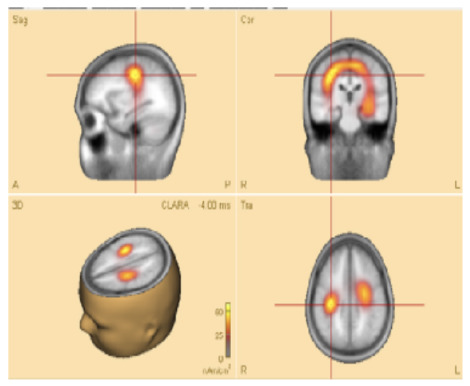
**P7**	**Male /24**	Tonic posture followed by clonic movements then evolution to bilateral tonic clonic seizure	Focal: frontal and right temporal lobe	Ictal/slow wave delta (T8, F8, FP2, FP1, F7)	Focal: starting at right temporal and extended into frontal lobe	Concordant	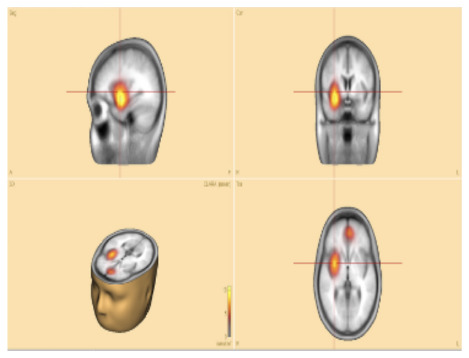
**P11**	**Male/41**	Staring attacks followed by tonic head and eyes deviation to right side then evolution to bilateral tonic clonic seizures	Multifocal: temporal, and parieto-occipital lobes (Multiple cavernoma)	Ictal/theta (T7, P7, P3, F8, T8, P8, O2)	Multifocal: temporal, parietal, and occipital lobes	Concordant	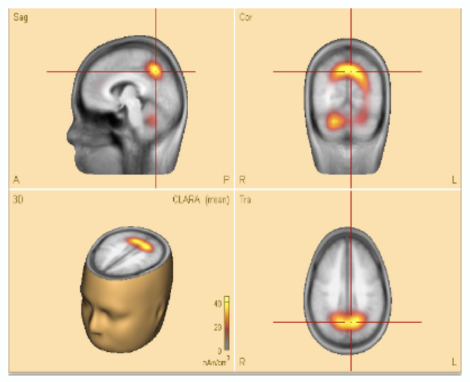

Each patient exhibited unique semiology. Classification was based on the literature (Blair, 2012; Fotedar et al., 2022; Jan and Girvin, 2008; Menghi et al., 2018) and the authors' clinical expertise. Fourteen patients were diagnosed with focal epilepsy and two with multifocal epilepsy. Among focal epilepsy cases, three patients had unilateral temporal lobe epilepsy (left or right). These temporal epilepsy patients underwent surgical intervention and were followed for at least 1 year postoperatively.

Of the 16 patients, 14 demonstrated clear, high-amplitude ictal and/or interictal epileptic features during 24-h EEG monitoring. These features were consistent across the recording period. They included regular or irregular slow delta, theta, alpha, beta, arrhythmic waves, and spike or spike–wave complexes. The expression of these features varied among patients. In two cases (patients 15 and 16), the ictal features were either too low in amplitude or masked by other EEG patterns, such as sleep activity. Thus, these patients were excluded from CLARA analysis.

When epileptic features stood out above baseline, the 2D voltage distribution maps for most waveform peaks within the pattern were uniform and consistent (see Figure 2). Using the averaged waveform from this pattern with an appropriate time window proved advantageous for source localization.

Analysis revealed that the first PCA component accounted for over 95% of the variance. Selecting the rising portion of the average waveform with optimized duration increased this effect. Focusing on this segment improved the precision of **SOZ** localization.

To assess seizure onset dynamics and identify potential propagation zones, CLARA was applied to multiple time points within the rising phase of the epileptic feature. This analysis revealed that in five patients (**P1**, **P2**, **P8**, **P9**, and **P10**), the **SOZ** remained consistent within a single lobe.

Three patients (**P4**, **P5**, **P11**) exhibited seizure onset zones (**SOZ**; regions where seizures originate) in multiple lobes, while six patients (**P3**, **P6**, **P7**, **P12**, **P13**) demonstrated SOZ with extensions into different lobes (Figure 3, bottom panel). For example, in patient **P7**, the initial **SOZ** and its subsequent propagation to other lobes could be tracked across different time points within the selected rising phase of the epileptic waveform. At the conclusion of the study, three of the 16 patients were excluded from the concordance analysis. Two patients (**P15**, **P16**) were excluded prior to CLARA computation (Combinatorial Localization Algorithm for Rapid Analysis; a computational tool for identifying seizure onset zones) due to the absence of a clear ictal pattern, and one patient (**P5**) was excluded after CLARA analysis because of a multifocal epileptic presentation with an indeterminate seizure onset. Among the remaining patients, the presumed and computed SOZ completely overlapped in 10 cases and partially overlapped in 3 cases, corresponding to a concordance rate of 77% and a partial concordance rate of 23%.

**Figure 4:**
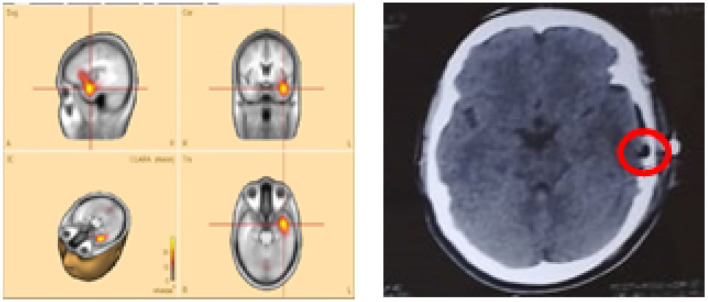
Sample **patient no. 8**. **(Left)** the CLARA area, and **(right)** the resected area in the post-surgical CT-scan.

To empirically validate the clinical relevance of the CLARA method (Combinatorial Localization Algorithm for Rapid Analysis), we examined the relationship between post-surgical outcomes and resection of the CLARA-identified area. Three patients who underwent lobectomy (surgical removal of a brain lobe) were available for retrospective analysis:

1. **Patient P11**—the CLARA-identified area corresponded to the center of the epileptic focus involving multiple cavernomas in the left temporal and parieto-occipital lobes. These lesions had been surgically resected several years earlier at our hospital. The patient has remained seizure-free with medication for more than 2 years. Retrospective analysis of pre-surgical VEEG confirmed that the CLARA-identified zone was located entirely within the previously resected region, illustrating a case in which surgical targeting of the CLARA area was associated with successful seizure control.
2. **Patients P2** and **P8**—both were initially evaluated at our hospital, with preliminary reports indicating left temporal epilepsy without further specification. Each subsequently underwent left temporal lobectomy at different centers, but neither experienced clinical improvement. Upon returning for follow-up, we applied the ESL methodology, which revealed that the CLARA-identified area was located in the left mesial and/or lateral temporal lobe. In both cases, surgical resection had been confined primarily to the lateral temporal lobe (Figure 4). The most plausible explanation is that the main epileptic focus identified by CLARA was either incompletely resected or missed entirely. However, other contributing factors, such as surgical technique, underlying pathology, or network-level epilepsy mechanisms, cannot be excluded.

Figure 4 illustrates **patient P8**, whose CLARA-identified zone was located in the left mesial temporal lobe near the temporal pole. Surgical resection (removal of brain tissue) was limited to the superior temporal gyrus of the lateral temporal lobe. Postoperative CT imaging confirmed only a small resection volume, and no clinical improvement was observed. Histopathological data were not available for this case.

## 4 Discussion

Pre-surgical brain mapping generally follows minimal guidelines. Neurologists typically rely on ictal semiology to identify the **SOZ**, while neurophysiologists use interictal and/or ictal EEG features to estimate its location. These approaches, however, remain inherently subjective and may provide insufficient precision for guiding surgical intervention.

There is no universally reliable semiology for all seizure types, particularly for extratemporal seizures. Moreover, epileptic discharges recorded on scalp EEG can spread rapidly across multiple channels, complicating the accurate determination of the **SOZ** and its potential propagation. As a result, reliance on semiology and visual EEG inspection alone can limit the accuracy of pre-surgical localization.

This study evaluated the performance of the CLARA method using BESA Research 7.1 software, which offers multiple electrical source localization (ESL) techniques. CLARA analyzes characteristic epileptic patterns to determine both the location and size of the seizure onset zone (**SOZ**). It can track EEG signal changes on a microsecond scale, enabling identification of the earliest epileptic activity and distinguishing the initial **SOZ** from its subsequent spread or extension.

The ictal and interictal features analyzed in this study likely originated from the epileptogenic zone. Accordingly, we focused on prominent epileptic features during the ictal period—such as slow waves, alpha waves, and other rhythmic or arrhythmic patterns—associated with at least three seizures. Interictal events, such as epileptic spikes or spike–wave complexes identified by physicians, were also analyzed. Incorporating both ictal and interictal features improves CLARA's ability to localize the **SOZ**.

In many patients, the CLARA-identified regions for ictal and interictal features overlapped within the same brain region, indicating robustness in identifying the **SOZ** across seizure phases.

The most definitive way to validate a new epileptic brain imaging technique is to compare its predicted SOZ with post-surgical outcomes following resection. In this study, one patient with multiple cavernomas underwent successful brain lobectomy, resulting in more than 2 years of seizure freedom. CLARA correctly identified the central area of the cavernomas in the left temporal and parieto-occipital lobes. In contrast, two other patients who underwent left temporal lobectomy at other hospitals experienced no clinical improvement.

Unfortunately, for these latter patients, no detailed records were available on the pre-surgical SOZ localization methods used. CLARA analysis showed that although both the CLARA-identified and resected regions were within the left temporal lobe, they did not fully overlap (Figure 4). This finding underscores the importance of accurate SOZ localization prior to surgery.

Neurosurgeons often take a conservative approach, such as removing a portion of the temporal lobe along with the amygdala and hippocampus in temporal lobe epilepsy cases. Ideally, surgical planning should aim for precise resection of only the epileptogenic tissue—whether temporal or extratemporal—while preserving as much functional cortex as possible. For example, in temporal lobectomy, ESL results can inform whether to:

1. Perform partial hippocampal resection (removing only the head) vs. complete hippocampal resection (extending to the tectal plate).
2. Resect or preserve the dominant superior temporal gyrus.

While the localization accuracy in this study was acceptable, there is room for improvement. To enhance the accuracy of ESL-based localization, we recommend:

1. Applying a conventional zero-phase bandpass filter (0.5 − 30 Hz) to extract epileptic features from the cleanest EEG segments, preserving maximum brain signal integrity without complex denoising.
2. Maximizing the number of repetitive waveforms with similar 2-D voltage distributions to improve the signal-to-noise ratio and obtain a representative average waveform.
3. Selecting an optimal segment between the onset and peak of the averaged waveform, ensuring the first PCA component explains (≥95%) of the variance for better source localization.
4. Co-registering the ESL solution with the patient's own MRI for realistic head modeling.
5. In advanced clinical research settings:

(a) Use high-density EEG (≥25 electrodes) for full-head coverage and improved spatial resolution.
(b) Co-register EEG data with patient-specific MRI, PET, and SPECT to achieve multimodal imaging and more precise SOZ localization.

In this study, the comparison was qualitative and restricted to the lobar level. We found minimal differences when comparing ictal and interictal CLARA zones, particularly when both were located in the same lobe. Therefore, further ictal-vs.-interictal comparison was not warranted for this dataset. However, future studies using high-density EEG (≥25 electrodes) and sub-lobar analysis may yield further insights.

This study has several limitations. The boundaries of the presumed and computed zones were not precisely delineated, which prevented a quantitative evaluation. As such, a qualitative concordance analysis was performed. Only 13 patients had usable data for concordance assessment, and only three had available post-surgical outcome information. This small sample size limits statistical power and generalizability, highlighting the need for further research in larger, more diverse cohorts.

Additionally, due to the lack of individual anatomical MRIs, CLARA source localization was performed using the standard MRI template provided by BESA. While this can reduce localization precision, especially for sub-lobar mapping, our primary analysis was at the lobar level, where the risk of significant mislocalization is lower. To improve anatomical accuracy, we used age-specific MRI templates rather than generic pediatric or adult models. These templates, in combination with FEM/BEM head modeling, cover a broad age range and reduce anatomical mismatch while maintaining feasibility across patient populations.

## 5 Conclusion

Unlike advanced epilepsy centers, many healthcare facilities lack access to comprehensive neuroimaging tools. Cost-effective, robust, and reliable ESL techniques could significantly improve the accuracy of seizure onset zone (**SOZ**) localization in these settings. The present study demonstrates a high concordance between the CLARA method and ictal semiology. This is further supported by favorable surgical outcomes in some patients following traditional lobectomy. Moreover, CLARA consistently identified the same brain regions across different epileptic features during both interictal and ictal periods. This reinforces its reliability for **SOZ** determination.

These results suggest that CLARA can serve as a valuable adjunct to clinical semiology and other neuroimaging techniques. This may enhance the precision of **SOZ** localization. In less advanced centers, integrating the ESL technique with the recommended steps outlined in this study may enable neurosurgeons to target specific epileptogenic regions, such as the mesial temporal or neocortical areas. This approach avoids relying on broader resections, such as traditional lobectomies. A more selective approach could maximize preservation of brain function while still achieving seizure freedom.

Although the CLARA technique itself is not novel, being an established feature of BESA software, the innovation of this work lies in its practical application within a resource-limited environment. Such settings are often characterized by restricted access to advanced imaging modalities (e.g., PET), reliance on low-channel EEG systems, and a shortage of specialized personnel. This study highlights the integration of CLARA with clinical semiology and video-EEG (VEEG) at the lobar level, providing a more objective and reproducible localization strategy. Importantly, we validated this approach against surgical outcomes, demonstrating its clinical relevance for presurgical evaluation where conventional resources are limited.

A promising future enhancement involves acquiring and analyzing ictal and/or interictal epileptic features during deep sleep. EEG recordings in deep sleep typically contain fewer artifacts and exhibit higher signal-to-noise ratios (SNR). Automated methods for detecting sleep phases in surface EEG, such as those described by Al-Bakri et al. (2018), could further refine the localization process. Adopting this approach has the potential to advance epileptogenic zone mapping and improve patient outcomes.

### 5.1 Study limitations

This study has several limitations that must be considered when interpreting the results. First, the small sample size limits generalizability and reduces statistical power. A larger cohort could validate the conclusions and make them more applicable across diverse patient groups. The lack of individualized MRIs for each participant is another limitation. Using a standard MRI template was needed due to the unavailability of individual scans. However, this may introduce localization bias and decrease the precision of source localization, especially at the sub-lobar level. Age-specific templates help address this, but it remains a constraint. Future studies should use individual MRIs when possible.

Finally, selection bias may also influence the results. The study used specific patient characteristics or inclusion criteria, which might limit sample representativeness. Future research should minimize such biases by including a more diverse set of participants and employing randomized selection methods where possible.

## Data Availability

The raw data supporting the conclusions of this article will be made available by the authors, without undue reservation.
